# Supplementary material for: The interactome of intact mitochondria by cross-linking mass spectrometry provides evidence for coexisting respiratory supercomplexes
Source: Mol Cell Proteomics. 2017 Dec 8;17(2):216–32. doi: 10.1074/mcp.RA117.000470 (PMC5795388; doi:10.1074/mcp.RA117.000470)
Supplement: Supplemental Data [file supp_RA117.000470_133922_0_supp_23978_fzffwf.zip › spectra_annotation/mito_DR_spectra_annotation/46-1-15-1-54-1.pdf]

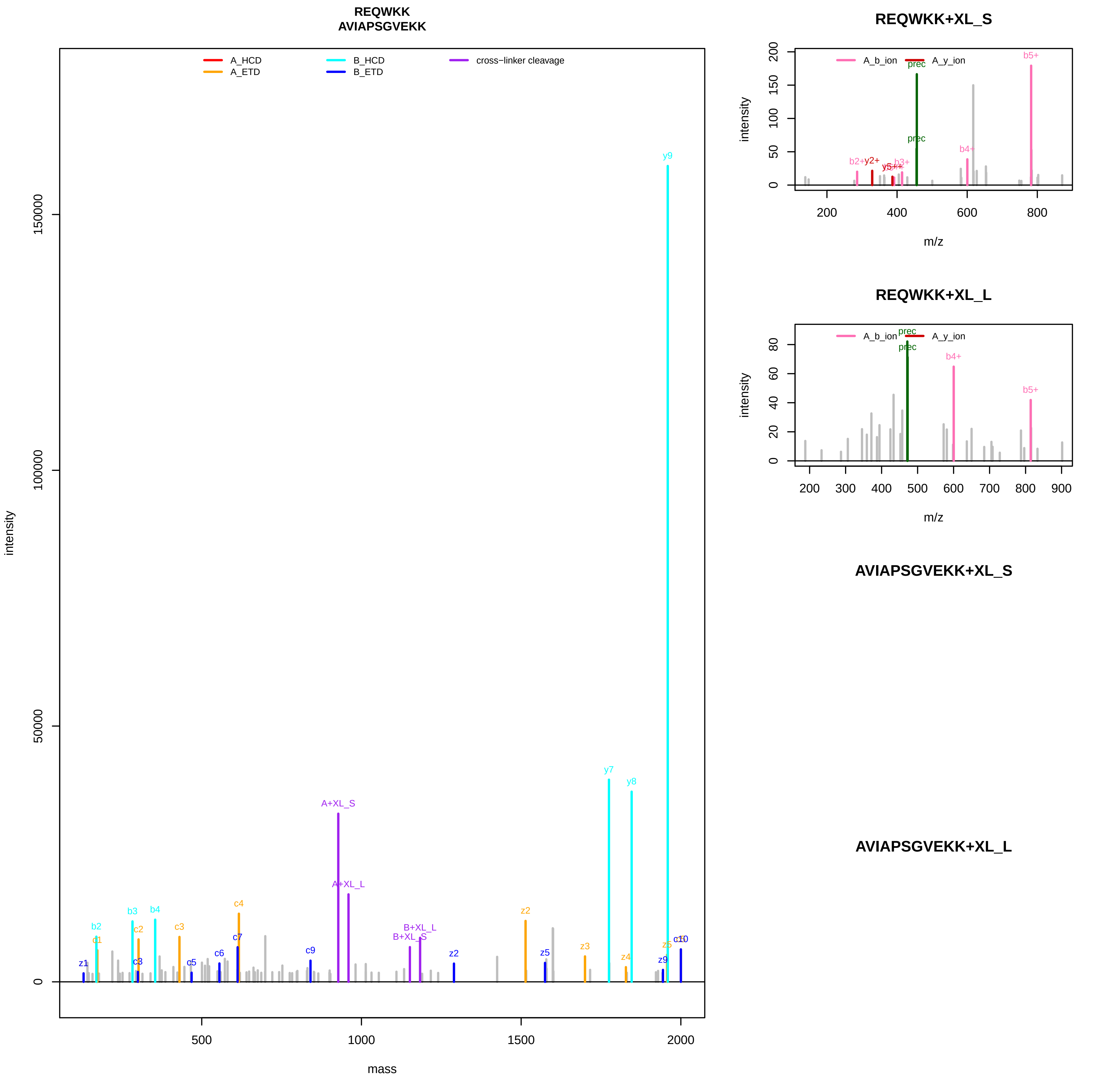
REQWKK
AVIAPSGVEKK
A_HCD
A_ETD
B_HCD
B_ETD
cross−linker cleavage
intensity
mass
150000
100000
50000
0
500
1000
1500
2000
y9
y7
y8
A+XL_S
A+XL_L
B+XL_L
B+XL_S
b2
b3
b4
c1
c2
c3
c4
c5
c6
c7
c9
c10
z1
z2
z3
z4
z5
z9
REQWKK+XL_S
A_b_ion
A_y_ion
prec
b2+
y2+
b3+
b4+
b5+
m/z
200
400
600
800
150
100
50
REQWKK+XL_L
80
60
40
20
300
500
700
900
AVIAPSGVEKK+XL_S
AVIAPSGVEKK+XL_L
